# Regulatory T Cells and Vitamin D Status in Children with Chronic Autoimmune Thyroiditis

**DOI:** 10.4274/jcrpe.2766

**Published:** 2016-09-01

**Authors:** Zeynep Şıklar, Deniz Karataş, Figen Doğu, Bülent Hacıhamdioğlu, Aydan İkincioğulları, Merih Berberoğlu

**Affiliations:** 1 Ankara University Faculty of Medicine, Department of Pediatric Endocrinology, Ankara, Turkey; 2 Ankara University Faculty of Medicine, Department of Pediatric Immunology-Allergy, Ankara, Turkey

**Keywords:** Treg cells, Chronic autoimmune thyroiditis, Vitamin D

## Abstract

**Objective:**

It is suggested that vitamin D is one of the factors that can regulate the function of Treg cells. In this study, the relationships between Treg cells and vitamin D levels was investigated in pediatric chronic autoimmune thyroiditis (CAT) patients.

**Methods:**

Thirty-two children with CAT and 24 healthy subjects were studied. FOXP3 expressing CD4+CD25+high Foxp3+T cells were identified as Treg cells. At diagnosis, 25-hydroxycholecalciferol (25OHD3) levels were determined in all patients. FOXP3 expression was measured before and after vitamin D replacement therapy in patients having low levels of 25OHD3.

**Results:**

In the CAT patients, Treg cell levels did not differ from the control group, while the frequency of vitamin D deficiency was higher and FOXP3 molecule expression was lower. FOXP3 molecule expression significantly increased in CAT patients having vitamin D deficiency who were given vitamin D replacement.

**Conclusion:**

FOXP3 expression is decreased in pediatric CAT patients. This reduction seems to be associated with vitamin D levels. Vitamin D can play a role in enhancing natural Treg cell functions.

**WHAT IS ALREADY KNOWN ON THIS TOPIC?**

It is suggested that vitamin D is one of the factors that can regulate the functions of Treg cells.

**WHAT THIS STUDY ADDS?**

In the pediatric patients with chronic autoimmune thyroiditis, reduction of FoxP3 expression seems to be associated with vitamin D levels. Vitamin D can play a role in enhancing natural Treg cells functions.

## INTRODUCTION

Chronic autoimmune thyroiditis (CAT) is the most common form of thyroiditis encountered in childhood and in the adolescent period (1). Similar to other autoimmune diseases, there are complex interactions between genetic susceptibility and environmental factors in the pathogenesis of CAT development. The loss of immune tolerance to self-thyroid antigens leads to autoimmune thyroid diseases (AITDs) (2). CAT is a T cell-mediated disease which is characterized by an activation of self-reactive CD4+ T lymphocytes, heavy T-cell infiltration in the thyroid gland, and progressive destruction of thyrocytes (3).

T cells have several subpopulations such as CD4+ helper, CD8+ cytotoxic, and regulatory T cells (Tregs) (4). Tregs are a specialized subset of T cells that are co-expressed as CD4+ and CD25+, which have a central role for immune tolerance by downregulating the inflammatory response against self-antigens (5,6,7). These cells exert their regulatory activity on CD4+ T cells, CD8+ T cells, B cells, and dendritic cells (4). One of the important characteristics of Tregs is the expression of the forkhead/winged helix transcription factor FOXP3 molecules that serve as keys in the maintenance of peripheral tolerance and in controlling the immune response (2). FOXP3 is a key gene in the development of Treg cells and it commits naive T cells to become Treg cells (4). Recent studies suggest that a decrease in FOXP3+Treg cells number and/or function is often associated with autoimmunity (8,9). While there is a growing number of studies on Treg cells in AITD in recent years (8,9), studies on autoimmune thyroiditis in humans are still limited. The exact role of Treg cells in the pathogenesis of CAT has not yet been fully recognized.

In recent years, increasing attention has also been drawn to the relationship between autoimmune diseases and vitamin D. Many cells of the immunological system, including T and B lymphocytes express vitamin D receptor and vitamin-D activating enzyme CYP27A1. Low levels of vitamin D were reported in patients with autoimmune diseases such as type 1 diabetes, lupus erythematosus, rheumatoid arthritis, and multiple sclerosis (2). Vitamin D is accepted as an immunomodulatory molecule. Some in vitro studies showed that vitamin D inhibits T cell proliferation. Vitamin D also appears to influence Treg cells differentiation and activity (10). Vitamin D stimulates the differentiation and activation of CD4+ lymphocytes, inhibits the differentiation of monocytes and dendritic cells and reduces the production of proinflammatory cytokines by Th1 cells (11). In addition, vitamin D may influence autoimmune disease risk and severity by dampening pathogenic Th17 cell IL-17 synthesis, amplifying a Th1–Tr1 switch (12).

Along with publications supporting the beneficial effect of vitamin D supplementation on autoimmunity, some studies could not demonstrate this effect (13,14). The situation with respect to CAT is not yet precisely clear.

As far as we know, the effect of vitamin D supplementation on Treg cells has not been demonstrated in children and adolescents with AITDs. In this study, aiming to contribute to clarification of the etiology of AITDs, we evaluated vitamin D status in CAT patients and in healthy children and adolescents in relation to the function of FOXP3+ Treg cells. We also investigated the possible changes in FOXP3 expression with vitamin D supplementation in vitamin D-deficient CAT cases.

## METHODS

The protocol for the study was approved by the Ethics Committee of Ankara University. Informed consent was obtained from all individual participants included in the study and their parents. The study was conducted between April 2013 and May 2014. Thirty-two children and adolescents with a diagnosis of CAT and 24 healthy sex- and age-matched subjects as a control group were enrolled to the study. The subjects in the control group were euthyroid and their were negative.

Inclusion criteria for both the CAT and control groups were as follows: 1) no experienced acute infection or any other illness during the 2 months period to the study; 2) absence of any other disease (hepatic, renal, immunological, etc.); 3) no medications including immunoactive drugs.

Clinical examination and assessment of pubertal status were performed in all subjects. The diagnosis of CAT was made by using conventional clinical, laboratory and ultrasonographic findings.

Blood samples were collected in the morning between 8:30 and 9:30 a.m. for serum levels of free triiodothyronine (fT_3_), free thyroxine (fT_4_), thyroid-stimulating hormone (TSH), antithyroid antibodies, 25-hydroxycholecalciferol (25OHD3) and for measurement of Tregs with FOXP3 expression. Determinations of TSH, anti-thyroid peroxidase antibody (anti-TPO), anti-thyroglobulin antibody (TgAb), fT_4_, and fT_3_ levels were performed by electro-chemiluminescence immunoassay (ECLIA) using Roche^®^ Elecsys reagent. Normal values ranged between 7 and 16 pmol/L for fT_4_, 3.8 and 6 pmol/L for fT_3_, and 0.34 and 5.6 mIU/mL for TSH.

The negative values for antithyroid antibodies were 0-4 IU/mL for anti-TPO and 0-9 IU/mL for anti-TG. The 25(OH)D levels were determined chromatographically using the isocratic high-performance liquid chromatography system.

Thyroid ultrasonography (US) was performed by an experienced radiologist. Subjects were examined with 7 MHz linear and an SSA 770 Aplio scanner (Toshiba Medical Systems Co, Ltd, Tokyo, Japan).

**Immunostaining and Flow Cytometric Analysis**

The frequency and precise number of Tregs were determined by three-color flow cytometry analysis performed on fresh whole blood collected in EDTA anticoagulant tubes. The blood cells were stained with antiCD4-PC5 (Beckman Coulter, Marseille, France), anti-CD25FITC (Beckman Coulter, Marseille, France), and intracellular anti-FOXP3-PE (eBioscience, San Diego, CA). Isotype-matched antibodies were used as negative controls. The blood samples were incubated at 4 °C for 45 minutes. After washing the cells twice with permeabilization buffer, fluorochrome-conjugated anti-human FOXP3 antibody (eBioscience, Cat. 12-4776. Clone PCH101) or isotype control were added. They were incubated at 4 °C for at least 30 minutes. Staining with mAb-anti-FOXP3 (antihuman FOXP3, eBioscience) was achieved according to the protocol recommended by the manufacturer with staining buffer set (eBioscience).

**Figure 1:**
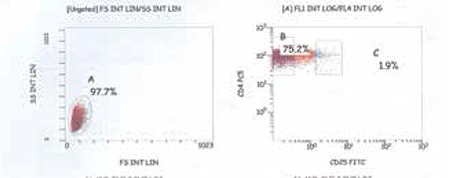
Gating strategy for frequency of Treg cells in the study population. Three color flow cytometry was performed in whole blood. Three subsets of CD4+T cells were defined according to CD25 staining cells expressing CD25 high were chosen and gated for the detection of FOXP3+T cells

Three subsets of CD4+ T cells were defined according to CD25 staining: CD25-, CD25^low^, and CD25^high^. Cells expressing CD25high were chosen and gated for the detection of FOXP3+ T cells (Figures 1 and 2a-2b). Three-color flow cytometry with Kaluza Software version was used for analysis by using NAVIOS^®^, Beckman Coulter, Miami. One single laboratory technician performed the flow cytometric analysis of Treg cells to avoid interindividual differences in technique.

Serum 25OHD3 levels below 20 ng/mL were considered as vitamin D deficient (15). Patients with low serum 25OHD3 levels were started on oral vitamin D3 (400 U/day) treatment. 25OHD3 levels were checked monthly. At least one month after the normalization of vitamin D levels, the percentage of Treg cells and FOXP3 expression were reanalyzed.

### Statistical Analysis

The Statistical Package for the Social Sciences (SPSS) for Windows 11.5 was used for statistical analysis. Student’s t-test, Fisher’s exact test, and Mann-Whitney U test were used to assess the differences between the groups. Wilcoxon signed ranks test was performed to compare CD4+CD25+^high^ T cell levels before and during treatment in the vitamin D-deficient patients. Statistical significance was considered when p < 0.05.

## RESULTS

In this study, 32 newly diagnosed CAT patients (aged 5 to 18.4 years) and 24 subjects as a control group were evaluated. The CAT (28 female, 4 male) and control groups (18 female, 6 male) were essentially similar with respect to age, height, and weight SDS. Although 7 (21.8%) of the patients in the study group had hypothyroidism, there was no statistically significant difference between the groups according to overall thyroid hormone levels (Table 1). Four of the hypothyroid subjects had compensated, while 3 had uncompensated hypothyroidism. There was also no significant correlation between thyroid hormone levels and the percentages of Treg cells in the CAT patients.

In the study group, the percentage of CD4+CD25+FOXP3+^high^ T cells (Treg cells) did not differ from that in the control group, while FOXP3 molecule expression was low (p=0.01). Vitamin D levels were also lower in the CAT group than in the control group (16.02 vs. 21.91, p=0.045). In the study group, there was a correlation between FOXP3 molecule expression and serum vitamin D levels (r=0.38, p=0.042).

The frequency of 25OHD3 D deficiency was higher in the study group as compared to the controls (68.7%-22 of 32 subjects) vs. 41.6%-10 of 24 subjects), respectively (p=0.04). Only one patient had hypothyroidism and normal vitamin D level on admission. Vitamin D levels were similar in the hypothyroid and euthyroid subjects (12.41 vs. 18.7 mcg/L, p=0.08).

Treg cells percentage and their FOXP3 molecule expression were reevaluated after replacement therapy in CAT patients with vitamin D deficiency when they achieved euthyroid status. Twelve CAT patients with low vitamin D levels were analyzed after vitamin D replacement. Vitamin D replacement was continued for 2 to 5 months (mean: 3 months).

The percentage of Treg cells did not change in the CAT patients with vitamin D deficiency who were given vitamin D replacement. On the other hand, FOXP3 molecule expression increased significantly (p=0.013). After vitamin D replacement, a statistically significant decrease in the level of thyroid antibodies was observed in CAT patients (Table 2).

Figure 1 shows gating strategy for frequency of Treg cells in the study population. FOXP3 expression of a patient with a low level of vitamin D at diagnosis and after vitamin D replacement is shown at Figure 2a-2b.

## DISCUSSION

Treg cells, by modulating potentially self-reactive T cells through secreting cytokines or by direct cell contact dependent mechanisms, play a crucial role in immune tolerance (7). The role of Treg cells in human autoimmune disease and in the pathogenesis of CAT is still not clear and under evaluation (16). It is suggested that patients with autoimmune diseases may have dysfunction or depletion of Treg cells (7).

In this study, the percentage of Treg cells and the expression of FOXP3 in children with a diagnosis of CAT was evaluated. The percentage of Treg cells in the CAT patients was found to be essentially similar to that of healthy children. However, there was a statistically significant reduction of FOXP3 expression in the CAT group when compared to the control group.

In a previous study, Ban et al (17) tested the FOXP3 gene locus for association with AITD in two large cohorts. They found no association between FOXP3 polymorphisms and AITD in the Japanese cohort, while there was a significant association in the Caucasian cohort. In another study, it was shown that the upregulation of Treg cells can suppress experimental thyroiditis (6).

The number of Treg cells was reported as adequate, increased, or decreased in AITD in several studies (8,9). Marazuela et al (8) showed an increase in the number of CD4+ CD25 T cells with molecular defect (a disturbed expression of IL-10, transforming growth factor-beta, genes for transcription factors FOXP3, STAT1, STAT3, and genes critical to Treg cells) in patients with AITD. They demonstrated that similar cells infiltrated into the thyroid tissue in CAT patients. These same authors concluded that the suppressive function of Tregs in peripheral blood was incomplete in these patients. An important study conducted on children with newly diagnosed AITD demonstrated a statistically significant reduction in the percentage of Tregs with the phenotype CD4+CD25^high^ and CD4+FOXP3 in children with AITD when compared to healthy children (9). In our study, patients with CAT showed a low FOXP3 expression compared to the control group, while Treg percentage was not different. The functional defect of Treg cells, similar to the low FOXP3 expression, could be specific for development of CAT. Either decreased number or impaired function of Treg cells may lead to the development of AITDs. Beside, increasing the FOXP3 level, upregulating the Treg cell functions may be an option for decreasing autoimmune responses in CAT patients.

Another aspect of our study was to evaluate vitamin D levels of the subjects and search for any relationship with Treg cells function, since Treg cells differentiation and activity can be influenced by vitamin D. Vitamin D has anti-inflammatory and immunomodulatory effects. In animal models, it was shown that administration of 1,25-dihydroxyvitamin D3 (or its analogs) arrests the immunological progression, thus preventing the clinical onset of autoimmune diseases (18). Indeed, vitamin D agonists have regulatory effects on the activation and differentiation of T-cells. It is also hypothesized that vitamin D deficiency can act as an environmental trigger that increases the occurrence of AITD (19).

Until now, the mechanisms underlining the role of vitamin D in thyroid autoimmunity are not completely understood. The relationships between thyroid autoimmunity and vitamin D have not been studied extensively. There are also conflicting results about the association of AITD with vitamin D levels. Although some papers do not confirm the connection between vitamin D levels and AITD, lower levels of vitamin D have been reported in several autoimmune diseases including Hashimoto’s thyroiditis and Graves’ disease (13,20,21,22).

Evliyaoğlu et al (23) have investigated vitamin D status in children and adolescents with CAT, and they found that CAT was observed 2.28 times more frequently in individuals with 25(OH)D3 levels <20 ng/mL. Camurdan et al (22) reported lower levels of vitamin D and higher vitamin D deficiency rates in children with Hashimoto’s thyroiditis when compared to the control group (22). In contrast, D’Aurizio et al (14) and Effraimidis et al (13) demonstrated that vitamin D levels were not lower in AITD patients than in controls. Effraimidis et al (13) carried a case-control study in subjects with normal TSH levels and no thyroid antibodies. During follow-up, cases with TPO antibody and controls with no TPO antibodies did not show any differences in their vitamin D levels. In the present study, we found a lower level of vitamin D in the study group than in the control group, also the frequency of vitamin D deficiency was higher in the study group on admission.

The cause of inconclusive results of low vitamin D level and AITD is not completely understood. Heterogeneous characteristics of study populations, levels of thyroid dysfunction, seasonal variability of blood sampling, the duration of disease, characteristics of the control groups, incidence of vitamin D deficiency in the population could be factors affecting vitamin D levels.

In our vitamin D-deficient CAT patients, after vitamin D replacement had been given, Treg cells percentage and FOXP3 expression were reanalyzed. We showed that vitamin D replacement could induce FOXP3 expression in children with CAT. With treatment, anti-TPO antibody titers showed a decrease. Although the number of cases is limited, this result gives a clue that vitamin D replacement can affect Treg cell function by increasing FOXP3 expression. For vitamin D treatment, we used the physiological replacement dose of 400 U/day. We do not know if higher doses of vitamin D would lead to more prominent changes in the percentage or function of Treg cells.

Although the number of patients is limited in our study, our results indicate an increased prevalence of vitamin D deficiency in patients with CAT. We based our findings on normal levels of vitamin D with respect to bone metabolism, even though these levels might vary for different organs or systems. In other words, adequacy of vitamin D levels for the immune system could differ from that for the skeletal system.

Factors such as some genetic and/or environmental changes could possibly affect FOXP3 expression. We do not know which of these factors are more effective in inducing the development of AITD. Hypothyroidism or LT4 treatment are additional factors which should have be taken into consideration. Actually, it has been suggested that LT4 therapy in hypothyroid patients can affect Treg cells functions (9). It has been shown that in hypothyroid patients, LT4 therapy leads to a decrease in IL-12, and it was suggested that a cytokine could be responsible for Th1 cell differentiation (24). In our study, there was no difference in serum vitamin D levels between hypothyroid and euthyroid patients in the CAT group. FOXP3 expression showed a similar increase in both euthyroid and hypothyroid CAT patients after replacement with vitamin D. These findings might confirm the immunomodulatory effects of vitamin D replacement. The decrease in anti-TPO titer in patients with vitamin D replacement is noteworthy. This finding might be reflecting the improvement of Treg cell functions of the patients after vitamin D replacement.

To conclude, in pediatric patients with CAT, FOXP3 molecule expression is decreased and this reduction appears to be associated with vitamin D levels. In patients requiring vitamin D, after replacement in physiological doses, the expression of FOXP3 molecules showed an increase. This result suggests that vitamin D can play a role in enhancing natural Treg cells functions.

## Ethics

Ethics Committee Approval: Ankara University Ethic Committe Approval Number 152-47-88, Informed Consent: It was taken.

Peer-review: Externally peer-reviewed.

## Figures and Tables

**Table 1:**
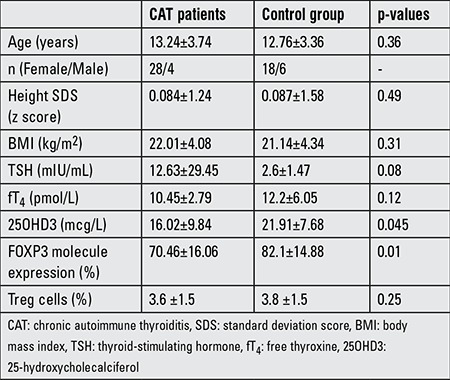
Characteristics of the subjects in the patient and control groups

**Table 2:**
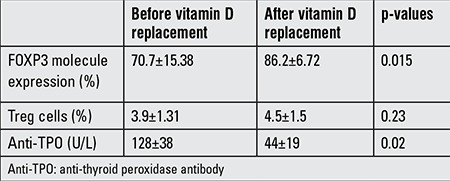
FOXP3 molecule expression and percentage of Treg cells in patients with vitamin D deficiency before and after vitamin D replacement

**Figure 2a:**
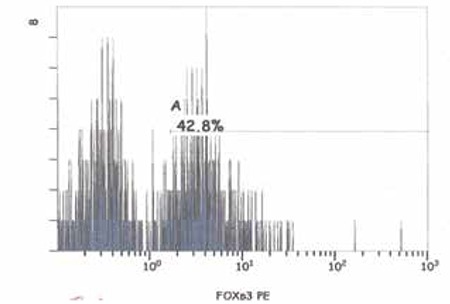
FOXP3 expression of a patient with a low level of vitamin D at diagnosis

**Figure 2b:**
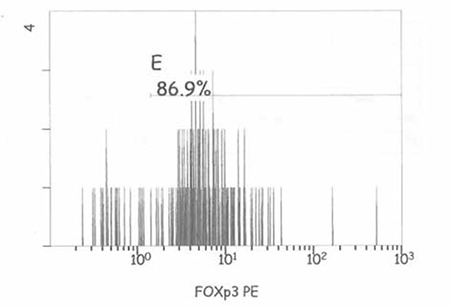
FOXP3 expression of a patient after vitamin D replacement
